# Nurse leaders' perceptions of leadership development needs for strengthening the nursing workforce: a South African pilot study

**DOI:** 10.3389/frhs.2025.1634563

**Published:** 2025-08-20

**Authors:** Mirriam Matandela, Goitsemang Lucy Chisale, Vhothusa Edward Matahela

**Affiliations:** ^1^ National Department of Health, Pretoria, South Africa; ^2^ Department of Health Studies, College of Human Sciences, University of South Africa, Pretoria, South Africa

**Keywords:** capacity building, health system strengthening, leadership development needs, nurse leaders, nursing workforce, South Africa, continuing professional development

## Abstract

**Background:**

Nurses are central to South Africa's healthcare system, yet the sector faces ongoing challenges, including workforce shortages, high workloads, limited career progression, and weak institutional support. These issues are exacerbated by the absence of a structured leadership development framework. In response, the national Department of Health, through the South African Nursing Leadership Initiative, launched a pilot study to explore nurse leaders' perceptions of leadership development needs prior to the development of a national Nursing Leadership Competency Framework. This study explored nurse leaders’ perceptions of the leadership capacity required to strengthen the nursing workforce and support national health priorities.

**Methods:**

A qualitative, exploratory study was conducted between May and July 2024, involving 153 purposively selected nurse leaders from four provinces. Data were collected through structured group narrative sessions, using semi-structured questionnaires aligned with global frameworks. A deductive thematic narrative analysis guided by Braun and Clarke's six-phase framework (2006) was employed to identify themes.

**Results:**

Three central themes emerged consistently across the pilot provinces. (1) *Workforce planning and career pathway development* revealed persistent nursing shortages and limited opportunities for career advancement. (2) *Capacity building through continuing professional development* highlighted the need for structured in-service education and leadership training to address skills gaps. (3) *Organisational support and leadership for retention* underscored high workloads, inadequate institutional support and the absence of psychological safety, all contributing to poor retention and morale.

**Conclusion:**

This pilot study provides contextual insights into the leadership development needs of a limited group of nurse leaders, which will inform the refinement of data collection tools and the design of a larger, national study. The findings are not generalisable but offer valuable direction for developing a contextually grounded Nursing Leadership Competency Framework and supporting strategic leadership capacity-building aligned with South Africa's health system strengthening goals.

## 1 Introduction

Effective leadership is essential in healthcare, particularly in nursing, where professionals operate in complex, high-pressure environments (1). Nurse leaders are pivotal in shaping the workforce, influencing patient outcomes and driving organisational performance (2). In South Africa, however, their effectiveness is challenged by high attrition rates, the migration of skilled professionals and ongoing resource constraints, particularly in rural and underserved areas (3). These systemic issues, compounded by the country's quadruple burden of disease and inequities between public and private healthcare (4), demand strategic, resilient and visionary nurse leadership.

Nursing leadership is increasingly recognised as a key driver for achieving national health priorities, including those articulated in South Africa's universal health coverage framework, known as the National Health Insurance (5). Effective leadership extends beyond administrative competence to include influence in workforce planning, institutional culture and policy advocacy (6). Yet, many nurse leaders lack access to structured, consistent leadership development, especially in resource-limited settings (7). Although frameworks and university-based programmes exist, their reach is often uneven. Globally, this reflects a broader trend: the World Health Organization's *State of the World's Nursing 2020* report cites a global shortfall of 5.9 million nurses, urging investment in nursing education, job creation, leadership and policy development.

In the African context, regulatory constraints, limited financing and role ambiguity hinder workforce growth, prompting calls for clarity in professional roles and better working conditions through the WHO's *Global Strategic Directions for Strengthening Nursing and Midwifery* (8). South Africa exemplifies these challenges, with an estimated nursing deficit of over 30 000 in the public sector and a vacancy rate of 15.7% (9). Nurse leaders must navigate institutional, political and socio-economic divides while fostering innovation and responsiveness (10). Their ability to drive system-level improvements is limited by underinvestment in leadership development. While initiatives in South Africa such as the RESON project (11) the primary care (PC101) (12), and clinical leadership hubs (13) have aimed to build capacity, expand nurses' roles and improve patient care, the overall impact has been mixed, reinforcing the need for sustained investment in strategic leadership, education and policy reform.

Addressing South Africa's quadruple burden of disease, alongside disaster and pandemic preparedness, requires empowered nurse leaders who can guide teams, influence policy and integrate technologies to strengthen healthcare delivery. However, there remains limited empirical insight into how nurse leaders perceive their own development needs or how these perceptions influence their capacity to respond to workforce challenges. Understanding these perspectives is essential for designing targeted, and contextually relevant interventions that support workforce retention, improve care quality and advance national and global health goals (14).

This pilot study explored South African nurse leaders' perceptions of their leadership development needs within a dynamic and resource-constrained health system. The pilot specifically sought to identify key themes, challenges, and priorities as expressed by nurse leaders, with the aim of informing the design and methodology of a forthcoming, larger national study. Conducted as the initial phase of a broader, multi-phase initiative by the national Department of Health to develop a Nursing Leadership Competency Framework, this article presents findings from the pilot phase, focusing on the dimension of *Strengthening the Nursing Workforce* within the larger competency framework development process.

## 2 Methods

**Figure 1:**
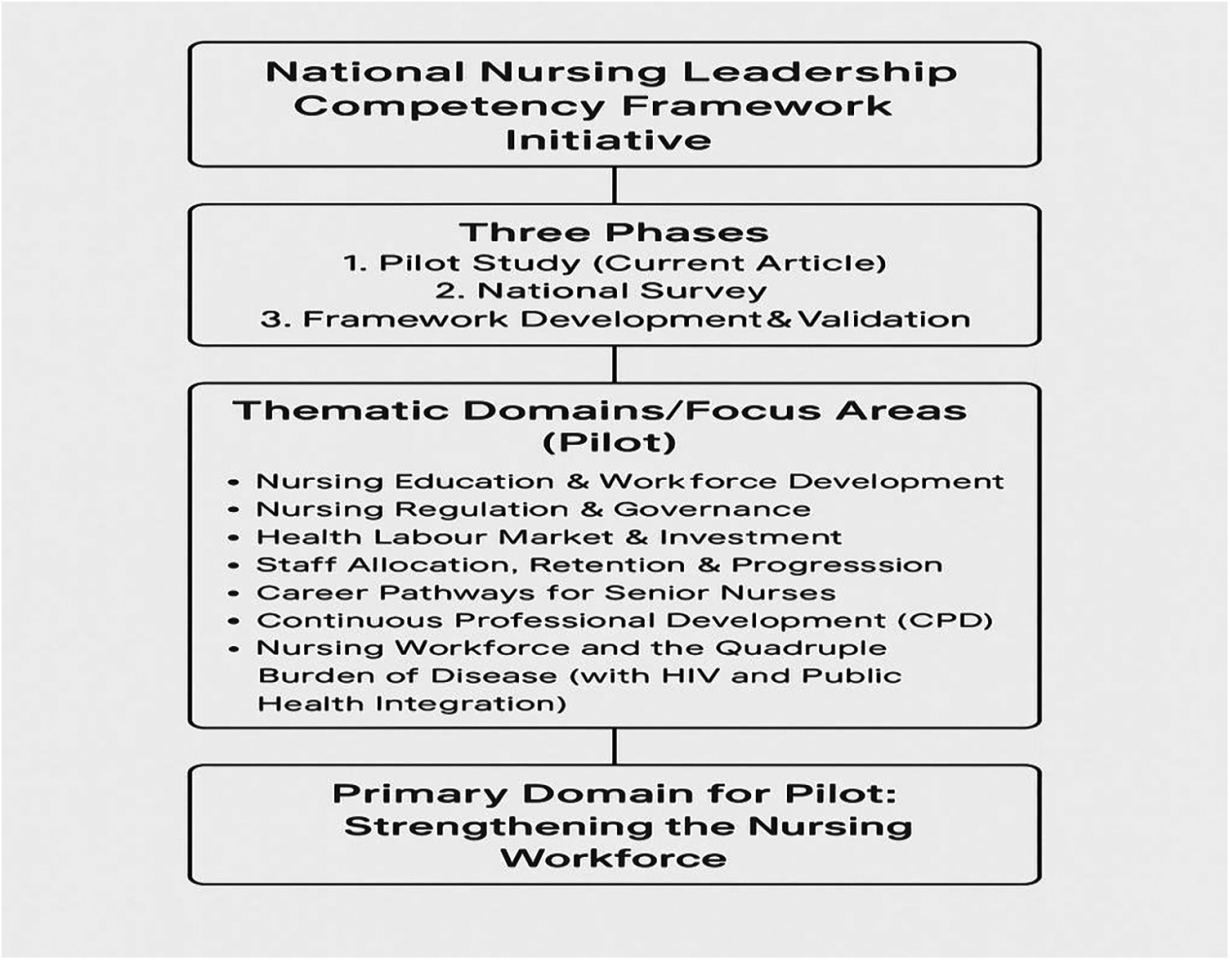
Overview of the national nursing leadership competency framework initiative. The diagram illustrates the three main phases of the initiative: the pilot study phase (current article), the national survey, and framework development and validation. The pilot study focused on seven thematic domains, including nursing education and workforce development, regulation and governance, labour market and investment, staff allocation and retention, career pathways for senior nurses, continuing professional development (CPD), and the nursing workforce in relation to the quadruple burden of disease. The primary domain addressed in the pilot phase was strengthening the nursing workforce.

This study forms part of a larger, multi-phase initiative led by the South African National Department of Health to develop a Nursing Leadership Competency Framework. The overarching initiative comprises three main phases (see Figure 1): (1) a pilot phase to explore leadership development needs and refine data collection tools; (2) a national survey phase to comprehensively assess leadership competencies among nurse leaders across all provinces; and (3) a framework development and validation phase to synthesize findings and establish a nationally relevant Nursing Leadership Competency Framework. Within this broader initiative, several thematic domains have been prioritized to address key leadership and workforce challenges.

For the pilot phase, participants were organized into seven thematic focus groups, each aligned with a core domain of the structured questionnaire: (1) gaps and best practices in nursing education and workforce development; (2) nursing regulation and governance; (3) health labour market and sustainable investment in nursing; (4) staff allocation, retention, and career progression; (5) career pathways for senior nurses; (6) continuing professional development (CPD); and (7) nursing workforce considerations in responding to the quadruple burden of disease, such as the integration of HIV and other public health services. Among these, the domain of *Strengthening the Nursing Workforce* served as the primary focus for this pilot phase.

This domain guided both the selection of discussion topics and the interpretation of findings, ensuring that the leadership development needs identified were closely linked to strategies for building and sustaining a robust nursing workforce in South Africa. By foregrounding this domain, the pilot provided critical insights to inform the subsequent phases of the initiative and the ultimate development of a contextually relevant competency framework. The structure of the study, including the pilot's thematic domains and the focus on Strengthening the Nursing Workforce, is depicted in Figure 1.

### 2.1 Study design and population

This study forms part of a larger, multi-phase initiative to develop a Nursing Leadership Competency Framework. The pilot phase adopted a qualitative, exploratory approach, conducted between May and July 2024, to explore nurse leaders' perceptions of leadership development needs for strengthening the nursing workforce. A total of 153 nurse leaders were purposively selected from four South African provinces (Eastern Cape, Free State, KwaZulu-Natal, North West).

Participants included senior nurse leaders from nursing education institutions, healthcare service settings, and provincial health departments, all holding formal leadership roles and actively engaged in workforce planning, professional development, policy implementation, and governance. District-level representatives were included to ensure diversity of perspectives across provincial contexts. Key informants were drawn from directorates including Corporate Services; HIV/AIDS, STIs, and TB (HAST); Human Resource Management and Development; Nursing Education and Practice; and various programmes such as Occupational Health and Safety (OHS), Infection Prevention and Control (IPC), Employee Assistance Programme (EAP), and Maternal, Child, and Women's Health (MCWH), as well as from Advocacy, Communication, and Health Information Systems. This ensured the engagement of stakeholders with specialized expertise in human resources, leadership development, infection control, health resources, and technology integration. Upon obtaining ethical approval for this study, participants were approached via official communication from provincial health authorities, informed of the study purpose, and invited to participate voluntarily. Informed consent was obtained from all participants, and confidentiality was assured throughout. Measures were implemented to minimize coercion and support self-determination, ensuring that participation had no impact on professional standing or employment (15).

### 2.2 Study setting

The pilot study was conducted across four of South Africa's nine provinces: Eastern Cape, Free State, KwaZulu-Natal, and North West. These provinces were purposively selected to reflect a diversity of health system contexts, with the intention of capturing a broad range of nursing leadership and workforce development challenges. The selection considered geographic, demographic, and health system variation, including provinces with substantial rural populations, logistical barriers to healthcare access, and regions with distinct experiences in managing the country's quadruple burden of disease.

The Eastern Cape, with its expansive rural areas, was selected because it provides perspectives on the unique leadership and workforce challenges in under-resourced and remote settings. The Free State and KwaZulu-Natal were selected because they bring about additional diversity: both have been at the forefront of nurse-led programme implementation and service delivery innovation, particularly in response to HIV/AIDS, which is a component of the wider quadruple burden that also includes non-communicable diseases, maternal and child health concerns, and injuries. North West was included to ensure further representation of provinces working to strengthen nurse-initiated care and address workforce gaps across multiple service areas. By selecting provinces with a mix of rural and urban characteristics, and with varying experiences in addressing public health priorities, the study aimed to capture the nuanced realities faced by nurse leaders across different provincial health systems. Thus, this approach supports the exploration of leadership development needs across the seven thematic areas, and contributes to a more comprehensive and contextually relevant foundation for the ongoing Nursing Leadership Competency Framework initiative. These contextual differences were essential for capturing a comprehensive understanding of workforce-related leadership development needs and for informing scalable, context-sensitive strategies to strengthen the nursing workforce nationally.

### 2.3 Recruitment of participants

Following the formal approval of a request by the Director-General of the national Department of Health to the provincial heads of health, nursing directors in the four pilot provinces purposively selected key informants to participate in the workshops. Although the target was approximately 40 participants per province, some individuals were unable to attend due to prior professional commitments. Despite this, participation remained strong, with final numbers as follows: Eastern Cape (*n* = 37), Free State (*n* = 33), KwaZulu-Natal (*n* = 40) and North West (*n* = 43). In the North West, three younger nurse leaders were intentionally included to support mentorship and succession planning, reflecting the broader aim of nurturing future nursing leadership. The sample size was considered adequate to achieve data saturation and generate meaningful insights to inform the development of a national Nursing Leadership Competency Framework. Participants were selected based on their leadership roles and relevant experience, ensuring the inclusion of knowledgeable informants capable of providing rich, context-specific perspectives on provincial leadership development needs.

### 2.4 Data collection approach

The pilot project was conducted in four purposively selected provinces, Eastern Cape, Free State, KwaZulu-Natal, and North West, between May and July 2024. Data were collected through in-person, face-to-face structured group narrative sessions held separately within each province. These sessions integrated focus group discussion techniques with the use of semi-structured qualitative questionnaires. The questionnaires were informed by globally recognised frameworks, including the *Global Nursing Leadership Competency Framework* (16), the *State of the World's Nursing Report* (17) and the WHO's (8) *Global Strategic Directions for Nursing and Midwifery* (2021–2025). A total of seven focus group discussions were conducted, one for each of the seven thematic areas identified in the structured questionnaire. Each focus group discussion took place within the respective province, with participants from that province only; at no point were participants brought together at a central, inter-provincial location. This decentralised, province-specific approach was used to ensure that discussions reflected the distinct contextual realities and leadership challenges present in each province. All narrative sessions were facilitated in person by trained members of the Nursing Leadership Initiative (NLI) project team, a collaboration between the national Department of Health and project funders. Facilitators used semi-structured prompts to encourage collaborative storytelling and discussion, enabling participants to express both personal and collective experiences related to leadership development. All sessions were audio-recorded with informed consent, and detailed field notes were taken to capture non-verbal cues, group dynamics, and contextual nuances. In addition to the verbal group discussions, participants provided written responses to open-ended questions either before or after the narrative sessions. These written responses were triangulated with the verbal narratives to enhance data richness and credibility. Participants were organised into seven thematic focus areas, corresponding to gaps and best practices in nursing education and workforce development; nursing regulation and governance; health labour market and sustainable investment in nursing; staff allocation, retention, and career progression; career pathways for senior nurses; continuing professional development (CPD); and nursing workforce considerations in responding to the quadruple burden of disease, including the integration of HIV and other public health services.

The pilot project was conducted in the four provinces between May and July 2024. Data were collected through structured group narrative sessions, which integrated focus group techniques with semi-structured qualitative questionnaires. These questionnaires were informed by globally recognised frameworks, including the *Global Nursing Leadership Competency Framework* (16), the *State of the World's Nursing Report* (17) and the WHO's (8) *Global Strategic Directions for Nursing and Midwifery* (2021–2025). This paper specifically presents findings related to the domain of Strengthening the Nursing Workforce.

Narrative sessions were facilitated by trained members of the Nursing Leadership Initiative (NLI) project team—a collaborative team from the national Department of Health and project funders. Facilitators used semi-structured prompts to encourage collaborative storytelling, allowing participants to express personal and collective experiences related to leadership development. All sessions were audio-recorded with informed consent, and field notes were taken to capture non-verbal cues, group dynamics and contextual nuances. In addition to the verbal discussions, participants provided written responses to open-ended questions either before or after the group sessions. These written inputs were triangulated with verbal narratives to enhance data richness and credibility.

Participants were organised into seven thematic focus areas, each reflecting a key domain of the structured questionnaire. These areas of inquiry included gaps and best practices in nursing education and workforce development; nursing regulation and governance; health labour market and sustainable investment in nursing; staff allocation, retention and career progression; career pathways for senior nurses; the extent of continuing professional development (CPD); and nursing workforce considerations in responding to the quadruple burden of disease, including the integration of HIV and other public health services.

### 2.5 Data analysis

All audio recordings from the pilot study were transcribed verbatim and anonymised to ensure confidentiality. The specific objectives of the pilot phase were: (1) to assess the feasibility and appropriateness of the structured group narrative methodology and data collection tools for the national study; (2) to identify key leadership development needs and challenges perceived by nurse leaders in diverse provincial contexts; and (3) to generate preliminary themes to inform the refinement of the forthcoming national Nursing Leadership Competency Framework survey.

Data from each province were initially analyzed separately to preserve provincial context and to identify unique local perspectives. Subsequently, datasets were merged for cross-case analysis, allowing the research team to synthesize both province-specific and cross-cutting themes. A deductive thematic narrative analysis approach was used, guided by the evaluation questions and predefined thematic categories from the structured tool, and structured using Braun and Clarke's six-phase framework (18).

The analytic process began with familiarisation, followed by systematic coding using both predetermined and emergent codes. Codes were grouped into broader categories to develop themes that reflected patterns of meaning across and within provinces. Emerging themes were collaboratively reviewed and refined by the NLI project team, ensuring internal coherence and alignment with the pilot objectives. Narrative synthesis enabled the construction of composite accounts reflecting collective experiences and diverse perspectives.

Validation of findings was achieved through member-checking: follow-up virtual or in-person sessions were conducted with participants from each province, during which preliminary results were presented for feedback. Participants were asked to confirm interpretations, suggest revisions, and prioritize leadership development needs identified in their context. This rigorous analytic approach ensured contextual sensitivity and reliability, providing a solid foundation for subsequent phases of the Nursing Leadership Competency Framework initiative. Notably, while the pilot study focused on refining methodology and surfacing initial leadership development themes, the overarching study seeks to develop and validate a comprehensive national framework for nurse leadership in South Africa.

### 2.6 Reflexivity and positionality

To enhance the trustworthiness of the research process, reflexivity was integrated throughout the study. The research team (NLI project team) comprised experts in nursing education, workforce policy, qualitative methodology and public health systems, including former and current nurse managers and academic leaders. Team members engaged in ongoing critical reflection through debriefing sessions, peer validation and interpretive dialogue to ensure that findings authentically represented participant experiences. Dissenting or divergent views were actively sought and integrated into the analysis to preserve the richness and complexity of the data.

### 2.7 Ethical considerations

Ethical approval for this pilot phase was obtained as an amendment to a previously approved protocol from the Walter Sisulu University Health Sciences Research Ethics Committee (Reference: 119/2024). The amendment covered the inclusion of additional sites and the specific focus on leadership development needs among nurse leaders. All participants provided written informed consent, and procedures were established to ensure confidentiality, voluntary participation, and the right to withdraw at any point without consequence.

## 3 Findings

A total of 153 nurse leaders participated in the pilot study across four provinces, as depicted in Table 1: Eastern Cape (*n* = 37), Free State (*n* = 33), KwaZulu-Natal (*n* = 40) and North West (*n* = 43). The majority of participants were aged between 51 and 60 years, particularly in KwaZulu-Natal and the Eastern Cape, indicating an experienced leadership cohort. Participants in the 61–70 age category were concentrated primarily in the Eastern Cape, while North West had the highest proportion of participants aged 30–40, suggesting some provincial variation in generational leadership representation.

**Table 1:** Participant demographics (*n* = 153).

Characteristic	Category	Eastern Cape (*n* = 37)	Free State (*n* = 33)	KwaZulu-Natal (*n* = 40)	North West (*n* = 43)
**Age**	30–40	0	5	0	11
	41–50	6	8	14	12
	51–60	18	18	24	20
	61–70	13	2	2	0
**Gender**	Male	10	8	8	27
	Female	27	25	32	16
**Designation**	CEO of hospital	2	1	1	2
	Chief Director	3	1	–	1
	Director	2	1	7	3
	Deputy Director	4	–	–	9
	Assistant Director	3	5	–	5
	Nursing Service Manager	5	–	–	2
	Deputy Manager: Nursing	5	–	6	7
	Principal	1	2	–	2
	Academic Head of Department	3	2	–	2
	Operational Manager	3	9	4	3
	Nurse/midwife	–	5	–	3
	Others	–	7	–	5
**Work setting**	Provincial office	7	2	12	9
	District	5	2	–	15
	Hospital	13	16	12	13
	Clinic/CHC	2	8	1	2
	Nurse education institution	4	5	9	4

Note: “–” denotes zero participants in the given category.

In terms of gender, the sample was predominantly female in the Eastern Cape, Free State and KwaZulu-Natal, whereas North West had a greater representation of male participants. This variation may reflect regional differences in gender representation in nursing leadership roles.

Participants held a range of senior positions across the nursing and health system hierarchy. Common designations included deputy directors, operational managers, assistant directors, nursing service managers and academic heads of departments. North West had a notably higher number of deputy directors and deputy nursing managers, whereas Free State had more operational managers. The category labelled “Others” captured participants whose roles did not align directly with the specified categories but who were integral to nursing leadership efforts.

Work settings included provincial offices, district management, hospitals, clinics/CHCs and nurse education institutions. Hospitals were the most represented work setting across all provinces, followed by provincial offices. KwaZulu-Natal showed stronger representation from nurse education institutions, and Free State had a notable proportion of participants working in Primary Health Care clinics and CHC settings.

Overall, the diversity in age, gender, role designation and institutional affiliation provided a comprehensive cross-sectional view of nursing leadership across the four pilot provinces, supporting the study's goal of gathering rich, context-sensitive insights to inform the development of a national Nursing Leadership Competency Framework.

Three key themes emerged from the analysis (see Table 2), each reflecting the leadership development needs and workforce challenges experienced by nurse leaders across the four pilot provinces: (1) *Workforce planning and career pathway development*; (2) *Capacity building through continuing professional development*; and (3) *Organisational support and leadership for retention*. These themes were consistently identified across all provinces and participant groups, with no notable differences observed by demographic characteristics or practice setting. It is important to note that, given the qualitative and deductive approach as well as the pilot scope, these findings reflect the perspectives of this specific group of participants and are not intended to be generalisable to all nurse leaders or settings in South Africa. Rather, the results provide valuable preliminary insights to inform the refinement of the national study and guide the ongoing development of the Nursing Leadership Competency Framework.

**Table 2:** Summary of major themes and sub-themes.

Themes	Subthemes
Workforce Planning and Career Pathway Development	- Persistent nursing shortages - Limited opportunities for career progression
Capacity Building through Continuing Professional Development	- Inadequate access to in-service education and leadership training
Organisational Support and Leadership for Retention	- High workloads and role strain - Lack of institutional support mechanisms

### 3.1 Theme 1: workforce planning and career pathway development

Persistent shortages of nursing personnel and constrained opportunities for career progression were consistently identified as key barriers to workforce stability and professional growth across the four provinces. These challenges were especially acute in rural and under-resourced settings, where shortages in specialised nursing cadres further limited service delivery and undermined long-term retention strategies.

#### 3.1.1 Sub-theme 1: persistent nursing shortages

Participants highlighted that international nurse migration and insufficient workforce investment continue to deplete the pool of experienced nurses, jeopardising health system resilience. The accounts reveal a strong perception that the South African health system lacks robust strategies for health labour market analysis and for retaining skilled nurses, particularly as other countries are seen as more attractive destinations. One participant underscored the challenge of international nurse migration, stating:

“Nurse leaders need to be developed in areas such as health labour market analysis and retention strategies to address the ongoing emigration of professional nurses. Countries like Australia, Saudi Arabia, and the UK continue to be seen as attractive by our nurses, and without sustainable investment and policy advocacy, we risk losing critical skills from our health system.” [P11 G4].

Another leader added, “The major gaps are the shortage of Professional Nurses and Specialist Nurses. There's an insufficient budget, and attrition posts aren’t being filled. We also lack nurses trained in key specialties. Right now, the estimated need to address the critical nursing workforce is around R600 million.” [P2 G2]. This quote reflects the financial and planning constraints currently undermining workforce sustainability. This dynamic is compounded by a lack of sustainable investment in the workforce, with leaders estimating a significant financial gap needed to address critical nursing shortages. A key insight from the data is that workforce shortages are not just a matter of numbers, but also of skills mix and service coverage. The integration of HIV services, for example, demands staff with highly specialised competencies and a strong commitment to patient engagement. Participants perceived that, although there is support for integrated care, the unique skills required for areas such as HIV management are not consistently developed or retained within the workforce.

One participant explained, “Although there is buy-in for the integration of services, managing HIV requires staff with a specific skill set, passion, and commitment. ART is lifelong treatment, and for the programme to achieve its objectives, those working with people living with HIV need the right acumen to build rapport and maintain patient trust.” [P34 G2]

Several participants pointed to a fragmentation between the education pipeline and clinical service, citing a mismatch between the numbers of nurses trained and those who find employment or suitable placements. For example, one noted, “There's a clear disconnect between nursing education and practice. This fragmentation affects the availability of clinical placements and contributes to the mismatch between the number of nurses trained and those actually absorbed into the workforce.” [P7 G3]. Thus, this gap not only restricts career progression but also weakens the pipeline for specialised roles and succession planning.

Another critical issue raised was the need for nurse leaders to be more effectively capacitated for strategic workforce planning. Participants argued that, without stronger leadership engagement in workforce forecasting and resource advocacy, nursing shortages are likely to persist:

“As nurse leaders we feel that there is a critical need to be capacitated so that we can advocate for increased training capacity and secure sustainable funding. Without leadership that can engage in strategic workforce planning and influence budget allocations, we will continue to underproduce nurses and struggle to meet healthcare demands.” [P7 G3]

This highlights the need for both immediate and long-term solutions to address workforce planning, including increased training capacity, improved alignment of educational outcomes with service needs, and more agile funding mechanisms.

#### 3.1.2 Sub-theme 2: limited opportunities for career progression

Limited opportunities for career progression were attributed to both institutional and systemic barriers. The lack of accredited nurse education institutions offering new specialty programmes was frequently cited as an obstacle, restricting nurses' ability to upskill or diversify their roles:

“Opportunities are limited, there is a lack of nurse education institutions accredited to offer the new specialty programmes, and that has blocked our career progression options.” [P5 G2]

Gender dynamics were also identified, with some participants observing ongoing biases and stagnation in women's advancement into senior leadership positions, while others described more equitable contexts. One participant observed, “The stagnation in the percentage of women in senior positions, despite ongoing appointments, suggests the limited opportunities for career progression among female health workers and points to persistent biases within leadership pathways.” [P24 G2]

Another participant said:

“No, in this province, more needs to be done to support women's leadership. For example, female leaders often face barriers to exercising decision-making authority equal to their male counterparts. Currently, only 14% of leadership positions are held by women.” [P11 P2]

In contrast, another participant from a different province offered a more positive view:

“I can say women in leadership are not discriminated against based on gender. Decisions are made in line with service delivery guidelines and the country's Constitution. Nurse leaders should be encouraged to sustain this culture of equity and ensure that supportive policies and practices continue to advance women's leadership across all levels.” [P12 G2]

Beyond gender, political interference and lack of professional autonomy emerged as persistent barriers. Participants shared that, when operational spaces are influenced by political connections rather than merit, this undermines the effectiveness of nurse leaders and impedes service delivery:

“Nurses are committed, ethical, and often embody servant leadership but political interference remains a major barrier. When politically connected individuals encroach on operational spaces, it undermines nurses’ ability to lead effectively and compromises service delivery. Nurse leaders need support to navigate these dynamics and advocate for professional autonomy within health systems.” [P38 G4]

The need for more effective succession planning was also emphasised, as current approaches are perceived to be inadequate for ensuring continuity of leadership as experienced professionals retire:

“Nurse leaders need to be capacitated in using HRH planning methodologies to maintain effective succession plans. Without this, we face ongoing leadership gaps and staffing challenges, especially as senior colleagues retire without clear plans for replacement.” [P35 G3]

Finally, motivation and retention of nurse leaders were strongly linked to remuneration and recognition. Despite the availability of some senior posts, insufficient compensation for leadership roles was seen as a significant demotivating factor, limiting the attractiveness of career progression and undermining long-term retention:

“Nurse leaders need to be developed in strategic human resource management to advocate for meaningful career pathways. While senior posts are available, the lack of competitive salaries for leadership roles limits motivation and undermines long-term retention.” [P19 G4]

These findings illustrate that workforce planning and career pathway development in South African nursing are shaped by a complex interplay of systemic shortages, training-practice mismatches, constrained career mobility, gender and political dynamics, and insufficient investment in leadership pipelines. Therefore, addressing these challenges will require not only technical solutions but also robust nurse leadership development, policy reform, and sustained resource commitment at all levels of the health system.

### 3.2 Theme 2: capacity building through continuing professional development

Limited access to structured, ongoing professional development remains a significant impediment to both individual nurse growth and broader health system responsiveness. Participants voiced a strong consensus that continuing professional development (CPD) is essential for equipping nurses to meet evolving clinical and managerial demands, yet described a landscape marked by inconsistent provision, lack of standardisation, and significant gaps in leadership training.

#### 3.2.1 Sub-theme 1: inadequate access to in-service education and leadership training

Across provinces, nurse leaders reported that opportunities for in-service education are often fragmented and dependent on the policies and resources of individual institutions. This disparity perpetuates inequities between urban and rural settings, with rural nurse leaders in particular describing more limited access to formal CPD and leadership development programmes. Nurse leaders emphasised that ongoing professional development is essential for addressing existing skills gaps, preparing nurses for advanced roles and enabling effective response to evolving healthcare demands. The absence of dedicated leadership pathways for nurses, distinct from generic skills development offered to all staff, was repeatedly highlighted as a missed opportunity for targeted capacity building. A participant shared:

“Currently, the department doesn't have specific leadership programmes for nurses. Nurse training is addressed under general skills development initiatives, but there is a recognised need for a dedicated leadership programme for nursing managers, yet this has not been developed or implemented.” [P39 G2]

Another explained, “The continuing development programmes are not standardised across the province—they largely depend on the individual institution's policies. If properly coordinated, these programmes could be powerful tools for building nursing capacity.” [P33 G3]

The data also illuminated persistent gender disparities in leadership development, with some participants observing a “glass ceiling” that limits women's access to senior leadership roles, while others noted emerging trends of increased male representation at higher levels:

“Although nursing is a predominantly female profession, we’re seeing a growing trend of males occupying leadership positions. The higher percentage of men in senior roles often diminishes the visibility, voice, and decision-making power of female nurses.” [P27 G2]

Participants consistently emphasised the growing importance of digital literacy and health technology integration for nurse leaders. While most nurses reportedly own smartphones and have basic digital skills, there is a substantial infrastructure gap within health facilities themselves, computers are scarce, and clinical areas often lack the technological tools needed to fully implement digital health solutions. One participant noted:

“Encouraging computer literacy as a compulsory course through CPD would help ensure that nurses already in the system aren't left behind by emerging technologies. This type of ongoing professional development is essential for building leadership capacity in a digitally evolving healthcare environment.” [P1 G3]

Thus, the digital divide not only hampers nurses leaders' ability to access and utilise data, but also limits the integration of digital competencies into CPD. Infrastructure limitations were also highlighted: “Nurse leaders need development in digital health planning to bridge the gap between personal device usage and institutional readiness. While most nurses, around 95%, own smartphones, facilities lack adequate digital health infrastructure, with computers often limited to reception areas and no tablets available for clinical use.” [P1 G4]

Educational leadership and the effective use of data in teaching were also highlighted as areas in need of further development. Some participants described resistance among nurse educators, particularly those from older generations, to adopting new technologies and evidence-based teaching methods. As a result, valuable clinical data and digital resources are underutilised in both training and practice, constraining the profession's ability to respond to changing service demands:

“There is a gap in how data is integrated into teaching. Nurse educator leaders struggle to support staff, especially older lecturers, who often resist adopting new technologies and teaching methods. Computer literacy and data analysis should be core parts of both the nursing curriculum and staff development, but at the moment, data from clinical practice isn't being used optimally because these competencies are missing in training.” [P30 G3]

These findings indicate that the capacity of South African nurse leaders to adapt to an evolving health landscape is shaped not only by the availability of professional development opportunities, but also by how well these programmes are tailored to local realities and emerging workforce needs. Addressing gaps in CPD, through standardisation, equitable access, leadership-specific curricula, digital health integration, and a focus on gender equity, will be crucial for cultivating agile, future-ready nurse leaders who can drive innovation and resilience in the health sector.

### 3.3 Theme 3: organisational support and leadership for retention

The analysis revealed that organisational support, encompassing workload management, institutional resources, and the psychosocial work environment, plays a decisive role in shaping nurse leaders' ability to foster staff well-being and drive retention. Across all provinces, participants described the cumulative pressures of high workloads, role strain, and limited institutional backing as pervasive threats to both individual morale and workforce stability.

#### 3.3.1 Sub-theme 1: high workloads and role strain

Participants consistently identified overwhelming workloads, exacerbated by shortages not only of nurses but also of essential support personnel such as clerks and porters, as a significant barrier to effective practice: “No, nurses are overworked not only because of nursing staff shortages, but also due to shortages in other essential support categories, like clerks and porters, which directly impact nurses’ workload.” [P23 G2]

Thus, the resultant role strain led to widespread reports of burnout, fatigue, and diminished motivation, particularly among those expected to balance frontline clinical responsibilities with leadership duties.

Importantly, leaders highlighted that excessive workloads directly undermine the capacity of nurses to stay current with best practices in infection prevention and control, and other priority areas. This, in turn, can compromise care quality, patient safety, and the overall resilience of the health system. A participant said:

“Due to high workloads and persistent staffing shortages, nurses often struggle to attend ongoing training. This limits their ability to stay updated on best practices in infection prevention and control. I think as nurse leaders we need to be empowered to create enabling environments that prioritise continuous learning without compromising service delivery.” [P13 G1]

Thus, the findings suggest that workload management must be recognised not only as a staffing issue, but also as a fundamental leadership challenge—one requiring nurse leaders to advocate for resource allocation, fair distribution of responsibilities, and the protection of time for staff development.

#### 3.3.2 Sub-theme 2: lack of institutional support mechanisms

This sub-theme emerged as a context-specific issue, varying significantly based on participants' work settings. Differences in geographic location, facility type and institutional resources shaped how nurse leaders experienced support or the lack thereof, highlighting disparities in access to mentorship, wellness services, digital infrastructure and professional development opportunities. A related insight was the impact of limited leadership representation and advocacy at higher levels of the health system. In some provinces, the absence of a Chief Nurse position or similar leadership posts was viewed as a structural barrier to ensuring that nursing perspectives are integrated into strategic decision-making and resource allocation. A provincial office participant stated:

“There is a need to develop nurse leaders who can advocate for stronger representation at the highest levels of the health system. In this province, we do not have a Chief Nurse position in the structure, only two Directors in the Nursing Directorate, so nursing perspectives are not fully integrated into strategic decision-making.” [P40 G4]

Another participant shared:

“Training resources do exist and help nurses build the skills needed to respond to current and future pandemics. However, limited mentorship and structural challenges, especially in rural areas, continue to hinder skill development and confidence in pandemic response.” [P39 G3]

Concerns around capacity and turnover were also raised:

“The healthcare system is overwhelmed by the volume of patients needing care. High clinical staff turnover leads to a brain drain, and without sustained institutional support, continuous training becomes a recurring necessity.” [P6 G3]

Psychological safety was also repeatedly identified as a critical but under-addressed aspect of organisational culture:

“Nurse leaders need to be capacitated to support staff who are reluctant to access internal wellness programmes. Many nurses feel uneasy using these services due to concerns about confidentiality and the fear that seeking help might affect how they are perceived professionally. Leaders must be equipped to recognise these concerns and create a culture of psychological safety within their teams.” [P15 G3]

“As nurse leaders, we need to start thinking about wellness advocacy and resource mobilisation to strengthen mental health support for staff. Because while services are available, often you will find that they are limited by inadequate staffing and infrastructural challenges that compromise accessibility and effectiveness.” [P4 G4]

This lack of trust and psychological safety was seen to hinder help-seeking, exacerbate stress, and ultimately undermine both staff well-being and retention. A participant noted the consequences of poor institutional support:

“Nurse leaders need to be equipped with skills in workforce management and staff well-being to address absenteeism and presenteeism. These issues are contributing to poor patient care, adverse events, and ultimately increasing the risk of litigation.” [P5 G4]

These findings indicate that strengthening organisational support for nurse leaders requires a multipronged approach, encompassing not only staffing and resource provision, but also the cultivation of supportive leadership practices, robust mentorship structures, and a culture of psychological safety. Addressing these areas is likely to be essential for mitigating burnout, reducing turnover, and ensuring the long-term sustainability of the nursing workforce in South Africa.

## 4 Discussion

This pilot study provides critical insights into the leadership development needs of nurse leaders across four South African provinces, focusing on strengthening the nursing workforce. The findings align with global literature that identifies effective nursing leadership as a cornerstone of resilient health systems, particularly in low- and middle-income countries where workforce constraints and systemic disparities are pronounced (8, 10, 13).

### 4.1 Workforce planning and career pathway development

Persistent nursing shortages emerged as a dominant concern among participants. Leaders consistently described chronic understaffing, especially in rural settings, and highlighted the impact of international nurse migration, lack of budget to fill vacant posts, and shortages in specialised cadres. These barriers are echoed in the literature, which cites migration, underinvestment in nursing education, and an aging workforce as key contributors to the global nurse shortage (12, 17). The need for leaders to be skilled in health labour market analysis and retention strategies, as voiced by participants, aligns with calls for evidence-based workforce planning and advocacy at policy level. Recent reforms in South Africa's nursing education sector, including the repositioning of public nursing colleges within the post-school education and training system and the introduction of competency-based curricula, have sought to address many of these structural barriers and align nurse training more closely with workforce needs (19).

Disconnects between nursing education and practice were also widely reported. Participants described fragmentation between academic and clinical settings, with limited availability of clinical placements and insufficient absorption of trained nurses into the workforce. This finding is consistent with studies advocating for improved integration of education and service, stronger mentorship, and clearer specialisation pathways to enhance readiness for practice (11, 20).

Limited opportunities for career progression were noted, with nurse leaders expressing concern about institutional barriers, lack of accredited education institutions for new specialty programmes, and inadequate remuneration for leadership roles. Some participants highlighted gender disparities and political interference as ongoing challenges, while others pointed to positive examples of gender equity and policy-driven leadership development. Succession planning was identified as an area needing urgent attention to ensure continuity as senior staff retire, reflecting recommendations for systematic leadership pipelines and sustainable workforce planning (10).

### 4.2 Capacity building through continuing professional development

Leadership development through CPD emerged as a central concern. Participants cited the absence of standardised in-service leadership programmes and uneven access to CPD, particularly in rural contexts. This finding aligns with global evidence advocating structured, context-specific CPD frameworks that address evolving healthcare demands and support lifelong learning.

The digital transformation of healthcare further amplifies the need for leadership in digital literacy. Participants emphasised that digital competency is now a critical leadership attribute, vital for data-informed decision-making and navigating complex systems (21). Moreover, gender equity issues were raised, with participants noting persistent barriers to the advancement of women into senior leadership positions. Despite some perceptions of equity, ongoing bias and limited visibility of female leaders call for intentional strategies to promote gender-transformative leadership (22, 23).

### 4.3 Organisational support and leadership for retention

The third major theme centres on the need for robust organisational support and leadership to retain the nursing workforce. Participants identified high workloads, poor infrastructure and fragmented wellness systems as key impediments to nurse well-being. These systemic issues, compounded by the absence of psychological safety and enabling work environments, contribute to burnout and attrition—echoing prior global findings (24).

The study also underscores the importance of people-centred leadership approaches that prioritise staff wellness, ethical work cultures and resilience. Leadership capacity must include competencies in managing presenteeism, mental health challenges and the emotional toll of care work (17, 25).

A particularly urgent area identified is the development of political astuteness among nurse leaders. Participants reported that political interference often undermines strategic leadership and nurse autonomy. Developing political acumen is crucial to enabling nurse leaders to influence policy, advocate professional interests and lead systemic reforms (26, 27).

Finally, the establishment of chief nurse roles at provincial level was strongly supported. These roles are essential for integrating nursing leadership in health governance and ensuring representation in policy development. As advocated by WHO (14), chief nurses must have the authority, resources and institutional access to drive quality, shape standards and align nursing with national priorities.

## 5 Implications for policy and practice

The findings support the urgency of developing and institutionalising a national framework for nursing leadership competencies in South Africa. Such a framework must prioritise leadership development in strategic planning, political astuteness, digital health, gender equity and workforce well-being. Nurse leaders must be equipped not only with operational and clinical leadership skills, but also with the capacity to influence policy, navigate power dynamics and advocate effectively for their profession. The creation of provincial chief nurse positions, as endorsed by WHO (14), is critical for embedding nursing perspectives in provincial governance and ensuring alignment with national health system goals. These recommendations are consistent with recent policy analysis, which emphasises the imperative of harmonising nursing education reforms with workforce needs, standardising recruitment and training processes, and fostering robust stakeholder engagement to advance the quality and sustainability of the nursing workforce (28). In line with these policy imperatives, future leadership programmes should be decentralised, contextually grounded, and responsive to both institutional realities and broader public health priorities.

## 6 Strengths and limitations

A key strength of this pilot study lies in its broad geographic and institutional reach, encompassing four diverse provinces and a cross-section of leadership roles within South Africa's nursing and healthcare system. This inclusivity enabled the collection of rich, context-specific insights that reflect both provincial variation and national leadership development needs. The use of structured group narrative sessions further allowed for collaborative sense-making among participants, enhancing the depth of data. However, limitations include the use of purposive sampling, which may restrict generalisability beyond the pilot provinces. As a qualitative study, findings are exploratory in nature but offer valuable foundational evidence to inform the development of a national Nursing Leadership Competency Framework.

## 7 Future directions

The findings from this pilot study provide preliminary insights into the leadership development needs of nurse leaders across four provinces in South Africa. Rather than offering national generalisations, these results highlight specific priorities and gaps within the participating provinces that warrant further exploration and validation in other contexts. Based on the pilot data, future directions for the next phases of the Nursing Leadership Competency Framework initiative include validating and refining the identified priorities through a national survey to determine whether the leadership development needs observed in this pilot are also reflected among nurse leaders in other provinces and settings.

There is a need to recognise the diversity of workforce challenges, such as rural staffing shortages, variable access to continuing professional development, and gender dynamics, which may necessitate tailored strategies for different provincial contexts. Additionally, methodological refinement is warranted, using lessons from the pilot to improve data collection tools, group narrative approaches, and member-checking processes for broader application. Further research should also examine the impact of specific interventions, such as digital health-focused CPD, succession planning programmes, and advocacy training, in both rural and urban settings and at various levels of the health system. Table 3 summarises the pilot-specific leadership development priorities, serving as a starting point for iterative framework development.

**Table 3:** Recommendations for the nurse leadership competency framework.

Thematic area related to strengthening the nursing workforce	Recommended nurse leadership competencies
a. Gaps and Best Practices in Nursing Education and Workforce Development	• Strategic planning for education-workforce alignment • Advocacy for integration of academic and service platforms • Curriculum evaluation and reform leadership • Monitoring and quality assurance of clinical placements
b. Nursing Regulation and Governance	• Policy advocacy and legislative literacy • Governance and accountability frameworks • Ethics, professionalism and regulatory compliance • Engagement with nursing councils and regulatory bodies
c. Health Labour Market and Sustainable Investment in Nursing	• Labour market analysis and workforce forecasting • Resource mobilisation and financial planning • Public-private partnership development • Political astuteness for intersectoral engagement
d. Staff Allocation, Retention and Career Progression	• Human resource planning and workload management • Retention strategy design and implementation • Equity-focused deployment models • Career pathway planning and mentoring
e. Career Pathways for Senior Nurses	• Succession planning and leadership pipeline development • Recognition of advanced clinical practice • Coaching and executive mentorship skills • Performance evaluation and talent nurturing
f. Extent of Continuing Professional Development (CPD)	• Lifelong learning culture promotion • Digital health and e-learning leadership • CPD framework development and implementation • Leadership in innovation and systems thinking
g. Nursing Workforce Considerations for the Quadruple Burden of Disease	• Integrated service delivery for HIV, TB, NCDs and MCH • Emergency preparedness and outbreak response leadership • Multisectoral collaboration and health diplomacy • Health promotion and population-based leadership

Priority should be given to future interventions that strengthen the alignment between nursing education and workforce needs, enhance governance structures and promote policy literacy and political astuteness. Additionally, the evolving health landscape necessitates investment in leadership capacities that address digital transformation, gender equity, interprofessional collaboration and multisectoral response to South Africa's quadruple burden of disease. Future efforts must also focus on standardising CPD to ensure equitable access, particularly for rural-based nurse leaders. The integration of digital health competencies and innovation into CPD programming will be critical to developing agile and forward-thinking nurse leaders.

Finally, advancing leadership succession planning, talent nurturing and structured career pathways for senior nurses will be essential to ensure sustained leadership capacity across all levels of the health system. These directions call for coordinated policy reforms, cross-sector partnerships and targeted capacity-building investments at institutional and governmental levels.

## 8 Conclusion

This study explored South African nurse leaders' perceptions of their leadership development needs as a foundation for strengthening the nursing workforce. Findings reveal critical gaps in career development, institutional support and leadership capacity, as well as in political astuteness, health systems planning and digital literacy. Addressing these needs through a national Nursing Leadership Competency Framework is essential for aligning nursing leadership with South Africa's health priorities to implement its universal health coverage framework effectively. Nurse leadership development must be viewed as a strategic investment in health system resilience. By empowering nurse leaders with the tools to lead policy, coordinate care and advocate for systemic reform, South Africa can cultivate a nursing workforce capable of navigating future challenges and achieving equitable, high-quality healthcare for all. This pilot served not only to gather early data on the leadership development needs of nurse leaders across diverse provincial contexts, but also to evaluate the effectiveness of the structured group narrative methodology and the thematic alignment of the questionnaire with national and global nursing leadership frameworks. The findings will contribute to the design of a more comprehensive study and ensure that the forthcoming competency framework is evidence-based, context-sensitive and aligned with South Africa's health system priorities.

## Data Availability

The raw data supporting the conclusions of this article will be made available by the authors, without undue reservation.
